# Parliament and Publications

**Published:** 1911-06-24

**Authors:** 


### Parliament and Publications.

### NOTIFICATION OF PULMONARY TUBERCULOSIS.

Replying to questions asked by Colonel Rawson and Mr. Raffan, the President of the Board of Trade explained that he had recently extended the range of notification of pulmonary tuberculosis, and that before proceeding further in that direction he proposed to await the results of that extension.

### POST-OFFICE SORTERS AND NEURASTHENIA.

Replying to Mr. Dawes, who asked how many sorters in the London postal service had died during each of the past five years as the result of mental and nervous diseases, Mr. Herbert Samuel replied that the number of deaths from these causes in 1906 was three, in 1907 nil, in 1908 four, in 1909 three, in 1910 nil. There were no figures available in his office of the number of officers who had received medical treatment for neurasthenia and kindred complaints.

### CERTIFICATES OF LUNACY AND IMBECILITY.

Mr. Lansbury asked the President of the Local Government Board if, in view of the fact that certificates in cases of lunacy and imbecility vary in cost from £3 13s. 6d. in St. George's-in-the-East to £36 14s. in St. Pancras per annum, he would consider the advisability of recommending some more uniform and economical method for dealing with these cases. Mr. Burns replied that the payments are made under statutory authority, but he was considering whether it is possible to take any action in the direction suggested.

### VACCINATION OF SOLDIERS' CHILDREN IN BARRACKS.

The Under-Secretary of State for War has been asked a number of questions relating to the vaccination of soldiers' children in barracks, the object being to ascertain whether the regulations under which children live in barracks "override the law of the land by requiring the vaccination of such children, irrespective of whether they have been exempted from the operation under Acts of Parliament." The replies to these questions may be summarised as follows : The residence in barracks of the families of soldiers is a privilege, and those granted the privilege must comply with the sanitary regulations for the use of the barracks; the residence in barracks of families who are unvaccinated is regarded as a danger to the health of the troops quartered there owing to the possible risk of infection.

### THE WORKMEN'S COMPENSATION ACT AND MEDICAL REFEREES.

Replying to a question asked in the House of Commons by Mr. Hancock, Mr. Churchill explained that medical referees under the Workmen's Compensation Act are not appointed for particular trades but for areas—namely, the County Court circuits and the districts into which the circuits are divided. In a number of circuits, specialist referees have been appointed for particular industrial diseases, such as lead poisoning, but subject to this the referees take all cases arising in their areas.

